# Multi-omics-guided HAMA/PLMA bioink integrating mechanical support, antibacterial protection, and liposomal kartogenin for cartilage regeneration

**DOI:** 10.1016/j.mtbio.2026.103546

**Published:** 2026-08-10

**Authors:** Tengfei Ma, Peixuan Wu, Yongning Sheng, Lian Duan, Jie Pei, Yanxin Liu, Kun Fu, Zeyu Liu

**Affiliations:** a Department of Joint Surgery, The First Affiliated Hospital of Hainan Medical University, Haikou, 570102, China; b Key Laboratory of Advanced Materials of Tropical Island Resources of Ministry of Education, School of Chemical Engineering and Technology, Hainan University, Haikou, 570228, China; c Department of Comprehensive Surgery, Hainan Public Health Clinical Center, Haikou, 570203, China; d Department of Surgery, Haikou People's Hospital, Haikou, 570208, China; e Department of Bioengineering, University of California, Los Angeles, Los Angeles, CA, 90095, United States; f Department of Joint Surgery, Hainan Sino-German Orthopedic Hospital, Haikou, 570200, China; g Center for Musculoskeletal Surgery, Charité–Universitätsmedizin Berlin, Berlin, 13353, Germany

**Keywords:** HAMA/PLMA hybrid hydrogel, 3D-printed bioink, Articular cartilage regeneration, Mechanotransduction, Antibacterial protection

## Abstract

Articular cartilage regeneration requires a bioink that combines printability with mechanical support, antibacterial functionality, and pro-chondrogenic bioactivity. Here, a 3D-printable hyaluronic acid methacrylate/polylysine methacrylamide (HAMA/PLMA, HP) hybrid hydrogel incorporating kartogenin-loaded liposomes (KGN@Lipo) was developed for articular cartilage regeneration. Integrated transcriptomic and metabolomic profiling identified HP-associated signatures related to ECM interaction and mechanotransduction, and selected mechanosensitive genes were subsequently validated by qRT-PCR. A second multi-omics comparison showed that KGN@Lipo@HP was associated with attenuated inflammatory–catabolic signaling and a matrix-protective cellular state relative to the Control condition. Physicochemical characterization confirmed rapid photocuring, reliable print fidelity, improved compressive behavior, cyclic resilience, and a tunable porous architecture, while liposomal loading preserved the optimized mechanical baseline. Functionally, KGN@Lipo@HP maintained high cytocompatibility, showed lower intracellular ROS-associated fluorescence, markedly inhibited *Staphylococcus aureus* growth, and promoted in vitro chondrogenesis, as evidenced by intensified cartilage-matrix staining and increased COL2 and ACAN expression. In the rabbit defect model, KGN@Lipo@HP was associated with the most extensive cartilage-like matrix deposition and the lowest histological scores among the tested groups. Compared with Control, the treated defects also exhibited distinct immunohistochemical profiles for cartilage formation and tissue remodeling. Together, these findings support an HP-based bioink platform that integrates mechanical support, antibacterial protection, and liposomal KGN bioactivity to support a cartilage-permissive regenerative microenvironment.

## Graphical abstract

**Figure:**
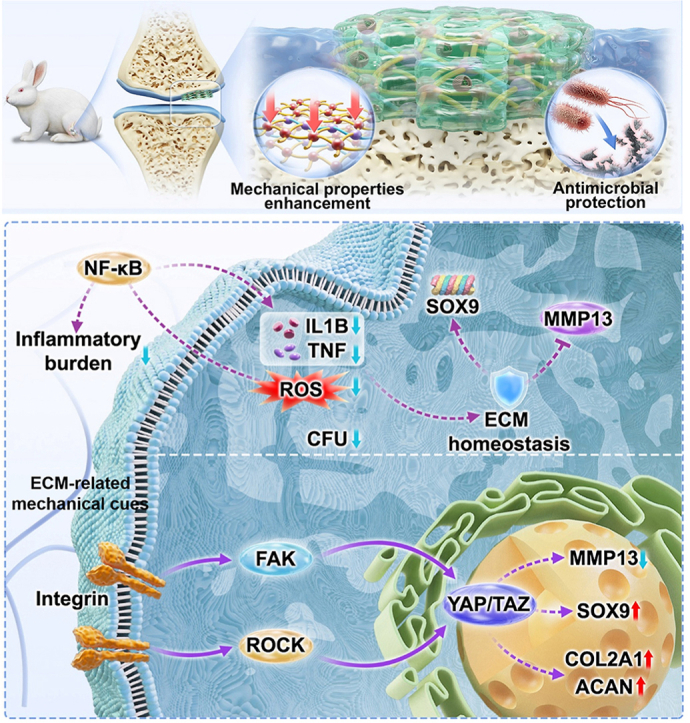

## 1 Introduction

Articular cartilage injuries remain difficult to repair because mature cartilage is avascular, alymphatic, and sparsely cellular, and therefore has limited intrinsic regenerative capacity after trauma or degeneration [[1], [2], [3], [4], [5]]. Current interventions can alleviate symptoms or partially fill defects, but they rarely restore durable hyaline-like tissue with native matrix composition, mechanical competence, and long-term homeostasis [[4], [5], [6], [7]]. This problem has shifted attention from simple defect filling toward biomaterial systems that can stabilize the lesion, regulate the local microenvironment, and direct anabolic remodeling.

Hydrogels are attractive in this context because their high water content and extracellular-matrix-like network architecture support cell accommodation and matrix deposition [[8], [9], [10]]. When coupled with 3D printing, hydrogel systems additionally offer spatial control, defect conformity, and programmable architecture [11,12]. Related studies in musculoskeletal regeneration have further demonstrated that 3D-printed scaffolds can integrate controlled therapeutic delivery, biomimetic lattice design, and hierarchical micro-/nanoporosity to coordinate structural performance with tissue-regenerative function [[13], [14], [15]]. Hyaluronic acid-based matrices are particularly suitable for cartilage applications because hyaluronic acid naturally occurs in both cartilage and synovial tissues [10,16]. However, hyaluronic acid and hyaluronic acid methacrylate (HAMA) matrices alone often lack sufficient post-cure mechanical strength and structural stability, which limits their performance as standalone printable bioinks [16,17]. A central design challenge is therefore to reconcile print fidelity, mechanical competence, and biological instructiveness within a single cartilage-regenerative construct.

Recent work in cartilage mechanobiology has further shown that an effective scaffold should be mechanically instructive rather than mechanically inert [[18], [19], [20]]. Chondrocytes interpret matrix mechanics through integrin-associated adhesions, cytoskeletal tension, and force-responsive transcriptional regulators, with FAK/ROCK signaling and YAP/TAZ-mediated mechanotransduction linking extracellular stiffness to cartilage gene regulation [19,21]. Mechanical support alone, however, is not sufficient in the injured joint microenvironment. During degeneration, oxidative stress, inflammatory signaling, and matrix-degrading enzymes tip matrix turnover away from anabolism and toward breakdown [[21], [22], [23], [24]]. At the same time, peri-implant bacterial contamination remains a practical but under-addressed risk in cartilage repair. Methacrylated polylysine-derived materials are attractive in this context because they combine intrinsic cationic antibacteriality with photocurability and 3D-printing compatibility [25]. Kartogenin (KGN) is a low-molecular-weight chondrogenic agent with documented cartilage-repair activity, but its limited aqueous solubility and short local residence time restrict direct administration [26]. Nano-enabled carriers can improve local bioavailability, protect hydrophobic cargo, and prolong bioactive presentation within hydrated matrices [27,28].

**Scheme 1:**
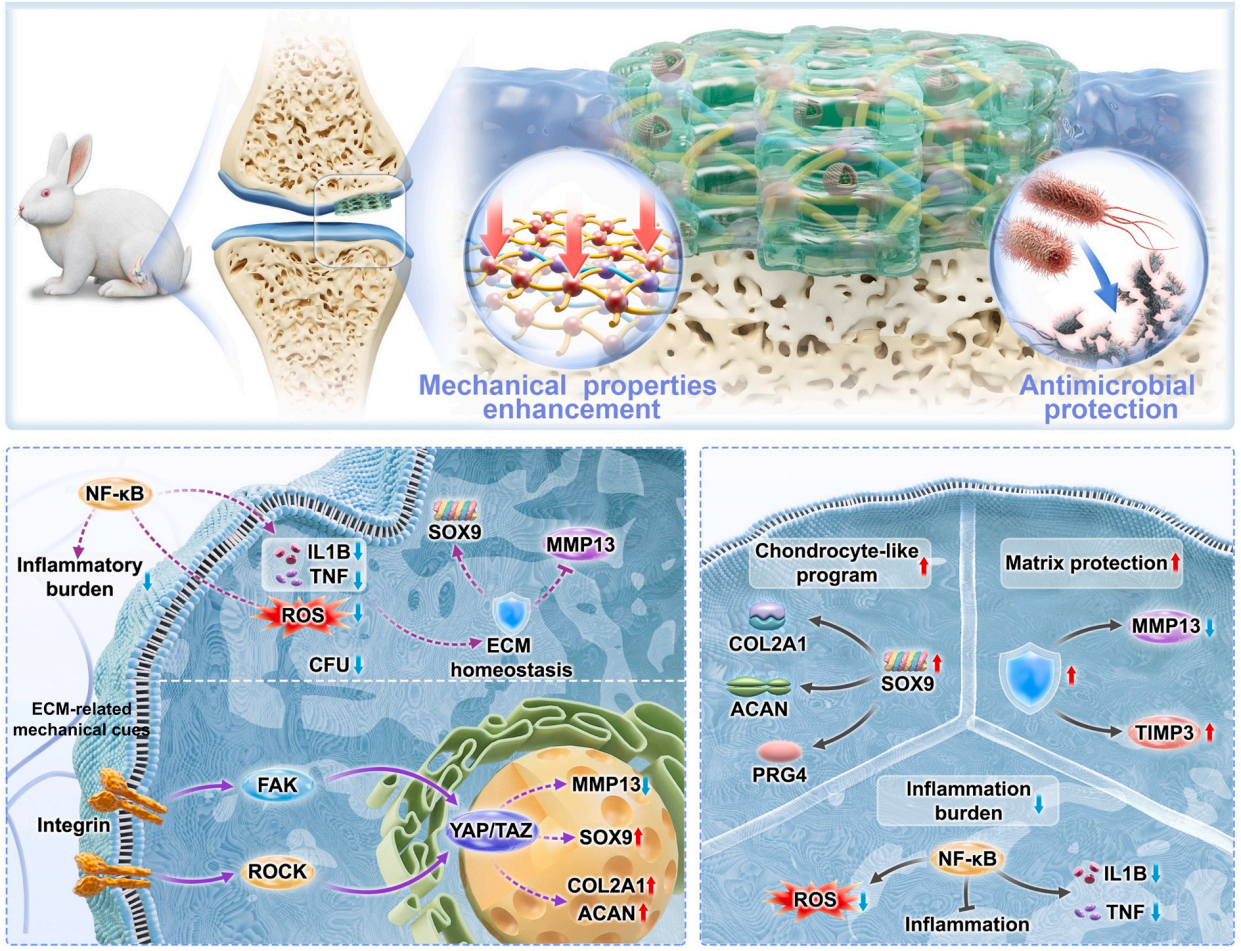
Design rationale and working model of the 3D-printed HAMA/PLMA hybrid hydrogel integrating KGN-loaded liposomes (KGN@Lipo) for articular cartilage regeneration. The printed construct combines a mechanically supportive extracellular-matrix-like network with intrinsic antibacterial functionality demonstrated in the tested assays. The proposed biological model summarizes an omics-supported association between extracellular matrix (ECM)-related mechanical cues and integrin/FAK/ROCK/YAP/TAZ-associated mechanotransduction signatures, together with SOX9-, COL2A1-, and ACAN-linked anabolic remodeling, reduced reactive oxygen species (ROS)-associated stress and NF-κB-related inflammatory signaling involving IL1B and TNF, attenuated MMP13-related catabolic signaling, and reinforced matrix protection.

Here, two linked scientific questions are addressed: whether a single 3D-printable bioink can simultaneously provide mechanical reinforcement, antibacterial protection, and sustained local KGN delivery while maintaining printability and structural performance; and whether these material functions are associated with systems-level cellular responses relevant to cartilage regeneration. To address these questions, we developed a HAMA/PLMA (HP) hybrid hydrogel incorporating KGN-loaded liposomes (KGN@Lipo). The innovation lies in integrating PLMA-mediated mechanical reinforcement and intrinsic cationic antibacteriality with liposomal KGN delivery, together with a staged multi-omics-guided evaluation of the HP-associated mechanotransduction baseline and the matrix-protective state associated with the final KGN@Lipo@HP construct. As summarized in Scheme 1, the platform was subsequently evaluated through formulation optimization, printing and physicochemical characterization, in vitro functional assays, and rabbit cartilage repair.

## 2 Materials and methods

### 2.1 Materials

PLMA, HAMA, and lithium phenyl-2,4,6-trimethylbenzoylphosphinate (LAP) were obtained from EFL (Suzhou Yongqinquan Intelligent Equipment Co., Ltd., Suzhou, China). Unless otherwise indicated, the remaining common chemical reagents were purchased from Macklin or Aladdin and used directly. iCell (Shanghai, China) supplied the commercial primary human bone marrow mesenchymal stem cells (hBMSCs; HUM-iCell-s011) and their matched medium (iCell-s011-002h). Penicillin-streptomycin solution (100×, C0222) was purchased from Beyotime. Sterile phosphate-buffered saline (PBS) and deionized water were used in all experiments.

### 2.2 Preparation and characterization of KGN-loaded liposomes

KGN@Lipo was formulated through thin-film hydration. Briefly, lecithin, cholesterol, and KGN were dissolved in chloroform according to the optimized KGN@Lipo formulation, with lecithin and cholesterol mixed at a mass ratio of 7:3. After removal of the organic solvent, a dry lipid film was formed and subsequently rehydrated with PBS under continuous stirring to generate the liposomal suspension. Unencapsulated KGN was removed by centrifugation. Blank liposomes were prepared in parallel using the same procedure without KGN.

Electron micrographs were used to assess morphology, whereas the DLS measurements yielded size-distribution and zeta-potential data for KGN@Lipo. Colloidal stability was evaluated by monitoring changes in hydrodynamic size and surface charge over time. Drug-release behavior was investigated by incubating KGN@Lipo in PBS, and the released KGN concentration was determined at predetermined time points using ultraviolet (UV) spectrophotometry at 279 nm.

Total and non-encapsulated KGN were quantified by UV spectrophotometry, and the resulting values were used to derive drug-loading capacity (DL) and encapsulation efficiency (EE). The amount of encapsulated KGN was calculated as: W_encapsulated_ = W_total_-W_free_, where W_total_ is the total KGN amount and W_free_ is the unencapsulated free KGN amount. EE was calculated as: EE(%) = W_encapsulated_/W_total_ ×100. DL was calculated as: DL(%) = W_encapsulated_/W_KGN@Lipo_ ×100, where W_KGN@Lipo_ represents the total mass of purified KGN-loaded liposomes used for the loading calculation. In this study, W_KGN@Lipo_ was calculated based on the KGN@Lipo formulation concentration of 0.2 mg/mL and the corresponding sample volume. Three independently prepared KGN@Lipo batches were analyzed, and each batch was measured in triplicate. All physicochemical data shown for this module were obtained from the liposomal system before incorporation into the hydrogel matrix.

### 2.3 Preparation of HP hybrid hydrogels and KGN@Lipo@HP

A 10 wt% HAMA solution in pH 7.4 PBS with 0.25 wt% LAP served as the photocurable precursor. PLMA was then added to HAMA to generate a series of hybrid formulations of HP containing 0%, 5%, 10%, and 15% PLMA. After complete mixing of HAMA and PLMA, uniform HP precursor solutions were obtained for subsequent testing. Based on the combined evaluation of compressive modulus, pore-size distribution, pore-size coefficient of variation, and cyclic compression behavior, the formulation containing 10 wt% PLMA was selected as the optimized HP hydrogel. Throughout this study, the PLMA content is expressed as mass fraction (wt%) in the precursor formulation.

For preparation of the bioactive formulation, KGN@Lipo was dispersed into the optimized HP precursor so that the final KGN concentration reached 100 μM, yielding KGN@Lipo@HP. Crosslinking was triggered under 405 nm light to generate stable hydrogel samples and scaffold constructs.

### 2.4 Projection-based photocuring 3D printing of scaffolds

An EFL-BP8601 pro bioprinter (EFL, Suzhou, China) fabricated the scaffolds for cell assays and animal implantation through a projection-photocuring workflow. The scaffold geometry comprised a 5-mm diameter, a 3-mm thickness, and 500-μm spacing between pores. HAMA, HP, and KGN@Lipo@HP scaffolds were prepared using the same printing and photocuring workflow. Printing was performed under 405 nm irradiation according to the optimized parameters summarized in Table S1. After printing and photocuring, the scaffolds were treated as described in Table S1 before biological assays or implantation. The printed and fully photocured scaffolds were used directly for material characterization and rabbit implantation or were used for cell-based in vitro assays.

### 2.5 Physicochemical characterization

Overall printing fidelity was quantified from binarized optical images using ImageJ and calculated as A_overlap_/A_model_×100%, where A_overlap_ represents the overlapping area between the printed scaffold outline and the predefined model. Pore-size distribution and pore-size coefficient of variation were quantified from scanning electron microscopy (SEM) images using ImageJ. At least 50 pores from three independent samples were analyzed for each group, and the coefficient of variation was calculated as standard deviation (SD)/mean × 100%. Lower values indicate more homogeneous pore architecture.

Uniaxial compression was used to obtain stress-strain curves and calculate the bulk compressive modulus, whereas cyclic loading was used to assess mechanical stability under repeated deformation. Samples prepared for SEM were freeze-dried and sputter-coated before microstructural imaging. The effect of increasing PLMA content on pore organization and structural uniformity was assessed from the SEM images. Fourier-transform infrared spectroscopy (FTIR) spectra of HAMA, PLMA, HP, and KGN@Lipo@HP were recorded to compare their chemical features. Swelling behavior was measured by immersing dried samples in PBS to equilibrium and calculating the swelling ratio from the corresponding mass change. Hydrogel degradation in vitro was followed gravimetrically. Each specimen was weighed once before PBS immersion at 37 °C (W0) and again at predefined intervals after retrieval and gentle rinsing (Wt). Residual mass (%) = (Wt/W0) × 100.

### 2.6 Cell culture

Primary hBMSCs (HUM-iCell-s011; iCell, Shanghai, China) at passages 3-5 were cultured in iCell-s011-002h medium containing 1% penicillin-streptomycin (Beyotime, C0222) under humidified conditions at 37 °C and 5% CO_2_. Unless otherwise stated, cell-based in vitro assays were performed by co-culturing hBMSCs with the corresponding 3D-printed and fully photocured HAMA, HP, or KGN@Lipo@HP scaffolds. A sterile PBS rinse and overnight equilibration in complete hBMSC medium preceded all cell exposures. After a 24-h attachment period at the assay-specific seeding density, one scaffold was positioned in a Transwell insert above each well; matched Control cultures received no scaffold. All cell-based experiments were performed using three independent hBMSC cultures per group.

### 2.7 Integrated transcriptomic and metabolomic analyses

Two integrated multi-omics comparisons were performed: Control versus HP and Control versus KGN@Lipo@HP. The Control-versus-HP comparison characterized the scaffold-associated mechanotransduction baseline, whereas the Control-versus-KGN@Lipo@HP comparison characterized the systems-level cellular state associated with the final construct. Later material characterization and functional assays included a dedicated HP-versus-KGN@Lipo@HP contrast to assess the added contribution of the KGN@Lipo module.

In each omics experiment, 2 × 10^5^ hBMSCs were seeded per well in six-well plates. After 24 h of attachment, cultures were exposed for 24 h to the corresponding HP or KGN@Lipo@HP scaffold, whereas Control wells remained scaffold-free. Cells were subsequently collected for total RNA and intracellular metabolite extraction. Each group included three independent biological replicates. NovaSeq sequencing generated paired-end reads of 150 bp for the transcriptomic analysis, and untargeted metabolic profiles were acquired by liquid chromatography-tandem mass spectrometry (LC-MS/MS). Genes were classified as differentially expressed when |log_2_ FC| > 1 and the adjusted p value was below 0.05. Differential metabolites were selected using the combined criteria of VIP > 1, Student's t-test p<0.05, and fold change ≥ 2 or ≤ 0.5. Data structure was examined by PCA and hierarchical clustering; pathway-level interpretation used Reactome, Kyoto Encyclopedia of Genes and Genomes (KEGG), and, where applicable, Gene Ontology (GO) enrichment, followed by integrated gene-metabolite association analysis. Gene–metabolite associations were assessed using Spearman correlation analysis. Detailed procedures for raw-data preprocessing, quality control, normalization, differential analysis, pathway-enrichment analysis, and transcriptome–metabolome integration, including software versions and analysis thresholds, are provided in the Supplementary Methods. These analyses were used to identify coordinated molecular signatures and generate testable working hypotheses, rather than to establish causality between individual molecular changes and subsequent functional outcomes.

### 2.8 Cytocompatibility, intracellular ROS, and qRT-PCR

hBMSCs were plated at 5 × 10^4^ cells per well in 24-well plates for viability assessment. Following 24 h of attachment, cultures were co-incubated for an additional 24 h with HAMA, HP, or KGN@Lipo@HP scaffolds; Control wells received no scaffold. Cells were incubated with 2 μM Calcein-AM and 4.5 μM propidium iodide for 30 min at 37 °C under light-protected conditions. All groups were then imaged with the Boshida BD-YGD-2 inverted fluorescence microscope using the same acquisition parameters.

The intracellular ROS assay used 24-well cultures containing 5 × 10^4^ hBMSCs per well. After 24 h for adhesion, each culture underwent a further 24 h of co-culture with the designated scaffold. After removing the scaffold and washing the cells with PBS, intracellular ROS-associated fluorescence was labeled with 10 μM 2′,7′-dichlorodihydrofluorescein diacetate (DCFH-DA) for 30 min at 37 °C in the dark. After removal of excess probe by washing, fluorescence images were acquired using the Boshida BD-YGD-2 microscope under identical imaging settings. For every biological replicate, three fields were chosen at random; their ImageJ fluorescence measurements were normalized to Control.

For gene-expression analysis, 2 × 10^5^ hBMSCs were plated in each well of a six-well plate. Following a 24-h attachment period, the scaffold assigned to each treatment group was placed in the corresponding Transwell insert, and co-culture was continued for another 24 h. Cellular RNA was then harvested with TRIzol™ Reagent (Invitrogen, Cat. No. 15596026), and first-strand cDNA was generated using Maxima™ H Minus cDNA Synthesis Master Mix (Thermo Scientific, Cat. No. M1681). Amplification was conducted in 20-μL reaction volumes containing SuperStar Universal SYBR Master Mix (CWBIO, Cat. No. CW3360H) on a CFX96 Touch Real-Time PCR Detection System (Bio-Rad). The thermal program consisted of 95 °C for 30 s, followed by 40 cycles at 95 °C for 10 s and 60 °C for 30 s; amplification specificity was subsequently assessed by melt-curve analysis. Two transcriptional panels were examined: ITGB1, PTK2, ROCK1, YAP1, WWTR1, and CCN2 for the mechanotransduction-associated response, and PCNA, CDK2, CDK4, and SOX9 for proliferation- and chondrogenesis-related responses. Expression values were normalized to GAPDH and reported as fold changes calculated using the $2^{-\Delta \Delta C_{t}}$ method. Each treatment group comprised three independent biological replicates, and the primer sequences are provided in Table S2.

### 2.9 In vitro chondrogenic evaluation and immunofluorescence

Each well in the 24-well chondrogenic assay received 2 × 10^5^ hBMSCs at passages 3-5. Following 24 h for adhesion, the cultures underwent 21 days in chondrogenic induction medium with HAMA, HP, or KGN@Lipo@HP; the parallel Control had no scaffold. Induction medium was renewed at 2-day intervals. On completion of induction, cartilage-matrix deposition was assessed by Safranin O and Alcian Blue staining.

Safranin O was eluted from one set of stained samples with 10% cetylpyridinium chloride and read at 492 nm. Separate samples were used for Alcian Blue quantification and immunofluorescence analysis. For Alcian Blue measurement, the retained dye was eluted with 6 M guanidine hydrochloride, and the absorbance of the resulting extract was measured at 620 nm on an RT-6100 microplate reader (Rayto, Shenzhen, China). For COL2 and ACAN immunofluorescence, samples were first fixed in 4% paraformaldehyde for 15 min and permeabilized with 0.1% Triton X-100 for 10 min. Nonspecific binding was minimized by incubation with 5% normal goat serum for 1 h at room temperature. Primary labeling was performed overnight at 4 °C using either rabbit anti-COL2 antibody (Abcam, Cat. No. ab34712; 1:200) or rabbit anti-ACAN antibody (Proteintech, Cat. No. 13880-1-AP; 1:200). Following washing, the samples were incubated for 1 h at room temperature in the dark with Alexa Fluor® 488-conjugated goat anti-rabbit IgG H&L (Abcam, Cat. No. ab150077; 1:500), and the nuclei were subsequently labeled with DAPI for 5 min. Fluorescence images were acquired using a Boshida BD-YGD-2 fluorescence microscope with identical acquisition parameters across all groups. For each biological replicate, fluorescence values from three randomly chosen fields were averaged in ImageJ to generate one replicate-level value; each group contained three independent replicates.

### 2.10 Antibacterial assay and infected rat cutaneous wound model

*Staphylococcus aureus* ATCC 6538 (FSCC223004; Guangdong Huankai Microbial Sci. & Tech. Co., Ltd., China) served as the challenge strain for antibacterial testing. To prepare scaffold extracts, fully photocured 3D-printed HAMA, HP, and KGN@Lipo@HP constructs were incubated in sterile PBS at 1:10 (w/v) for 24 h at 37 °C. Control PBS was incubated under the same conditions without a scaffold. Each scaffold extract or Control PBS was inoculated with bacteria to a final density of 1 × 10^5^ CFU mL^−1^ and incubated for 24 h. The suspensions were subsequently serially diluted and plated, and colony formation was assessed after 18 h at 37 °C. The resulting colony counts were used to compute bacterial killing as (CFU_Control_−CFU_sample_)/CFU_Control_×100%.

An infected rat cutaneous wound assay was used as an auxiliary in vivo assessment of the anti-infective feasibility of the KGN@Lipo@HP formulation. Randomization assigned adult male Sprague-Dawley rats (200-250 g) equally to Control and KGN@Lipo@HP, with three animals in each group. Under sterile conditions, a rectangular full-thickness dorsal skin wound was created and inoculated with *Staphylococcus aureus* at 1 × 10^6^ CFU per wound. The KGN@Lipo@HP group received the uncured KGN@Lipo@HP precursor formulation directly at the wound site every 2 days, whereas the Control group received no material treatment. Gross wound images were acquired at week 0 and at week 4, and wound areas were quantified using ImageJ. This auxiliary model was not intended to represent intra-articular infection or serve as a cartilage-repair endpoint.

### 2.11 Rabbit articular cartilage defect model and treatment

Approval for the animal procedures was granted under protocol HYMLL-2025-004. Under the approved institutional animal-welfare protocol, in vivo repair was assessed in rabbits bearing surgically created articular cartilage defects. Six-month-old male New Zealand White rabbits (approximately 2.5 kg) were randomly assigned to the Control, HAMA, HP, and KGN@Lipo@HP groups (n = 3 per group). The scaffolds used for implantation were fabricated and fully photocured before surgery according to the printing procedure described above. No additional in situ photocrosslinking was performed after scaffold placement in the cartilage defect. Once anesthesia had been induced and the surgical field prepared aseptically, one femoral condyle was prepared with a cylindrical defect measuring 5 mm across and 3 mm in depth. The defects in the treatment groups were filled with the preprinted and fully photocured scaffold constructs, whereas the Control group received no scaffold treatment. Animals received routine postoperative antibiotic and analgesic care. At 12 weeks after surgery, the animals were humanely euthanized, and specimens encompassing the defect region were harvested for histological staining and immunohistochemical analysis.

### 2.12 Tissue histology, immunohistochemistry, and scoring

Excised tissues underwent fixation, decalcification, paraffin embedding, and routine histological sectioning. The stained sections served three readouts: defect filling, tissue architecture, and cartilage-matrix deposition. The stain panel comprised H&E, Masson's trichrome, Alcian Blue, and Safranin O/Fast Green (SO/FG). Immunohistochemical detection targeted COL2, SOX9, RUNX2, and β-catenin with the corresponding primary antibodies, followed by standard signal development. Stained sections were imaged and subjected to semiquantitative analysis. Section- or field-level measurements were averaged within each rabbit before statistical testing, so that every animal contributed a single value. Representative fields shown in the figures were selected from the verified source images to reflect the corresponding group-level histological findings. Histological repair quality was assessed using Wakitani's and Sellers' scoring systems based on stained sections. Cartilage-like matrix fill was quantified from SO/FG-stained sections using ImageJ. The original defect region was delineated, and the Safranin O-positive repair area within this region was divided by the total defect area and expressed as a percentage. Section- or field-level measurements were averaged within each rabbit before statistical testing, so that every animal contributed a single value.

### 2.13 Statistics

Results are reported as mean ± SD. Unless otherwise indicated, each quantitative in vitro analysis was based on three independent replicates, with the experimental unit defined in the relevant Methods subsection or figure legend. Statistical analyses were performed in SPSS. For datasets involving two independent groups, significance was assessed using an unpaired two-tailed Student's t-test. Datasets comprising more than two groups were evaluated by one-way ANOVA followed by Tukey's post hoc test. In the rat cutaneous wound assay, the Control and KGN@Lipo@HP groups were compared independently at week 0 and week 4 using unpaired two-tailed Student's t-tests.

Each rabbit cartilage-repair group included three independent animals. No formal a priori power calculation was performed; the sample size was selected based on the exploratory proof-of-concept design, experimental feasibility, sample sizes reported in comparable cartilage-repair studies, and the principle of reducing animal use. A p value < 0.05 was considered statistically significant. NS denotes no significant difference; ∗, ∗∗, ∗∗∗, and ∗∗∗∗ denote p < 0.05, p < 0.01, p < 0.001, and p < 0.0001, respectively. Exact p values for selected quantitative comparisons shown in the main figures are provided in Table S3.

## 3 Results and discussion

### 3.1 Mechanical multi-omics of HP

**Fig. 1:**
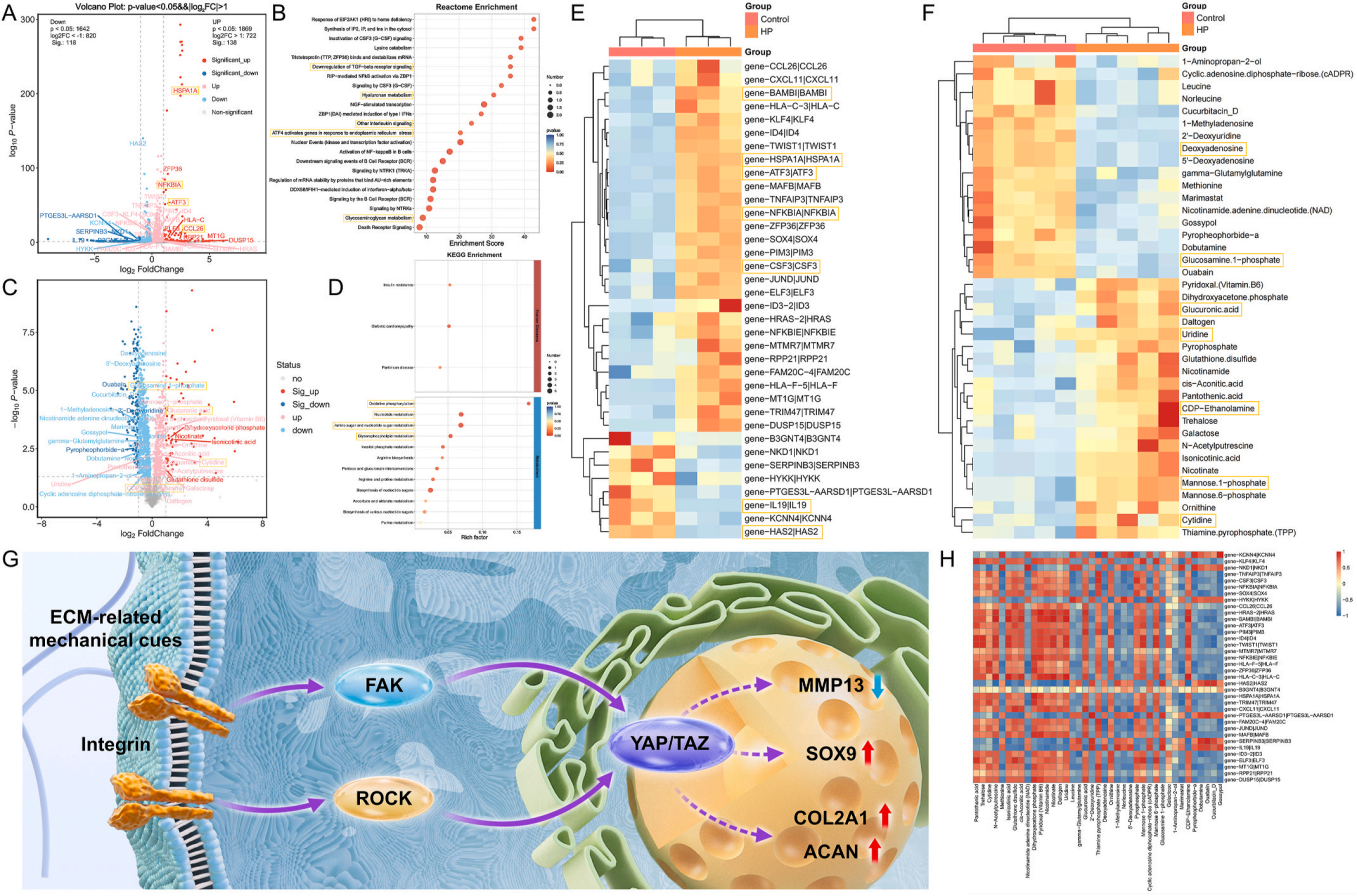
Mechanical multi-omics analysis of HP relative to the Control group. (A) Transcriptomic volcano plot comparing the Control and HP groups. (B) Reactome terms enriched among Control-versus-HP differentially expressed genes, with emphasis on HP-associated changes in ECM interaction, cytokine regulation, and stress-responsive pathways. (C) Control-versus-HP metabolite changes displayed as a volcano plot. (D) KEGG pathways enriched among metabolites that differed between Control and HP, illustrating the scaffold-associated metabolic response. (E,F) Hierarchical clustering heatmaps of representative differentially expressed genes (E) and metabolites (F). (G) Omics-supported and literature-informed working model linking HP-associated ECM-interaction and mechanotransduction signatures with putative downstream cartilage-anabolic and catabolic remodeling. Solid arrows denote literature-established canonical relationships within the mechanotransduction framework, whereas dashed arrows indicate proposed downstream relationships not directly tested in the Control-versus-HP comparison. (H) Integrated gene–metabolite correlation heatmap showing coordinated associations between differential genes and metabolites in the Control and HP groups. Selected genes, metabolites, and pathway terms discussed in the text are highlighted in the corresponding panels for visual reference.

To establish a systems-level biological framework for the mechanically reinforced HP scaffold, we first characterized the cellular response associated with HP relative to the Control condition using integrated transcriptomic and metabolomic analyses. The transcriptomic volcano plot showed marked gene-expression divergence between the two groups (Fig. 1A). Reactome enrichment further linked these changes to TGF-β signaling, hyaluronan metabolism, cytokine-associated pathways, and stress-responsive processes, consistent with HP-associated remodeling of matrix-interactive, cytokine-related, and stress-responsive cellular programs (Fig. 1B).

Metabolomic analysis revealed a similarly distinct response. In the HP group, the metabolomic volcano plot indicated widespread changes (Fig. 1C). KEGG pathway mapping connected these metabolite shifts with glycerophospholipid metabolism, nucleotide metabolism, amino sugar and nucleotide sugar metabolism, oxidative phosphorylation, and related biosynthetic routes (Fig. 1D), a pattern compatible with broad adaptation to the scaffold. Hierarchical clustering of representative genes and metabolites further separated the Control and HP groups at both the transcriptomic and metabolomic levels (Fig. 1E and F). Supplementary metabolomic analyses further supported this result, with principal component analysis (PCA), expanded enrichment, and additional clustering consistently indicating a robust metabolite-level response to HP (Fig. S1).

Fig. 1G summarizes an omics-supported and literature-informed working model in which the HP-associated ECM-interaction and mechanotransduction signature is linked to a proposed downstream shift toward SOX9-, COL2A1-, and ACAN-associated anabolic remodeling and away from MMP13-associated catabolic signaling [4,5]. These downstream nodes are included to contextualize the proposed direction of cartilage-related remodeling and are not interpreted as directly validated outputs of the Control-versus-HP comparison. The integrated transcriptome–metabolome correlation matrix showed coordinated associations between transcriptional and metabolic remodeling (Fig. 1H), while the expanded association network and heatmap in Fig. S2 illustrated broader gene–metabolite relationships. To further examine this mechanotransduction-associated signature at the transcriptional level, qRT-PCR analysis of representative mechanosensitive markers was performed in hBMSCs. To determine whether the HP-associated mechanotransduction signature was maintained after KGN@Lipo incorporation, the same marker panel was additionally examined in the HAMA and KGN@Lipo@HP groups. Markers were chosen to represent ECM sensing, focal-adhesion signaling, Rho/ROCK-dependent cytoskeletal tension, YAP/TAZ-regulated transcription, and subsequent ECM remodeling; Table S4 details the selection rationale. HP increased the expression of ITGB1, PTK2, ROCK1, YAP1, WWTR1, and CCN2 relative to the Control and HAMA groups (Fig. S3). For PTK2, ROCK1, YAP1, and WWTR1, expression in KGN@Lipo@HP remained comparable to that in HP, indicating that KGN@Lipo incorporation did not materially alter these mechanotransduction-associated transcriptional markers. ITGB1 and CCN2 were further increased in KGN@Lipo@HP, indicating additional transcriptional changes in markers related to cell–matrix interaction and ECM remodeling.

Together, the Control-versus-HP multi-omics analysis and qRT-PCR validation support a mechanotransduction-associated signature in HP-treated hBMSCs. This interpretation is consistent with emerging evidence that matrix mechanical cues can influence MSC aging and regenerative competence [29]. More broadly, recent studies indicate that stem-cell mechanotransduction involves both canonical integrin–cytoskeleton–YAP/TAZ signaling and non-canonical mechanically responsive pathways [30,31]. Accordingly, the integrin/FAK/ROCK/YAP/TAZ-related pathway is presented here as a supported working model rather than a fully resolved causal mechanism. Future studies using independently tunable matrices, pathway-specific perturbation, and dedicated MSC-aging readouts will help define stiffness-specific signaling mechanisms in greater depth.

### 3.2 Physicochemical and mechanical validation of HP

**Fig. 2:**
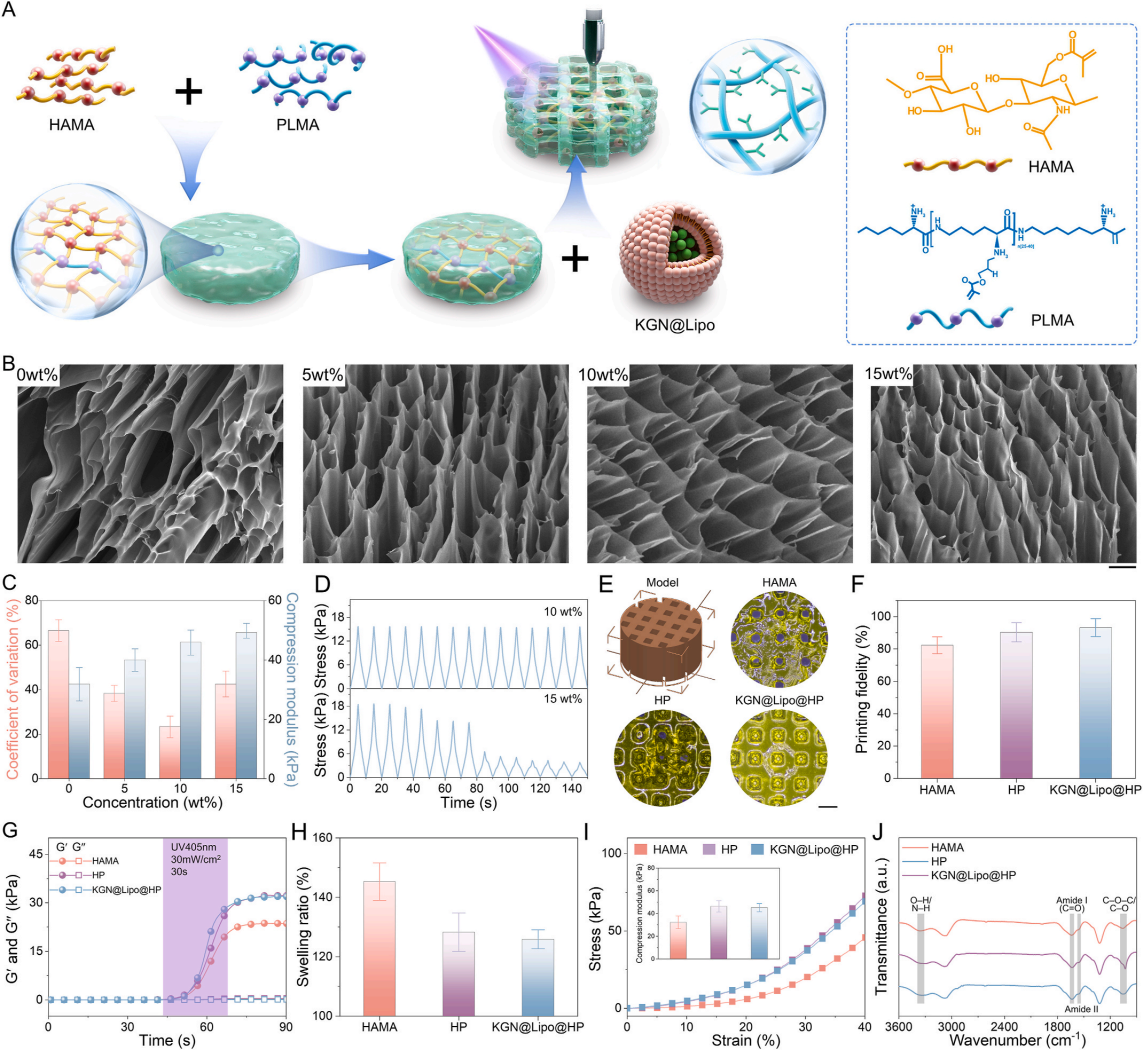
Formulation optimization, printability, and physicochemical characterization of the HP-based bioink. (A) Schematic illustration of the hybridization of HAMA and PLMA to form HP, KGN@Lipo incorporation, and fabrication of KGN@Lipo@HP by projection-based photocuring 3D printing; the chemical structures of HAMA and PLMA are shown on the right. (B) SEM images of photocured hydrogels with increasing PLMA mass fractions, showing PLMA-dependent changes in the lamellar porous microstructure. Scale bar, 20 μm. (C) Quantitative comparison of compressive modulus and pore-size coefficient of variation for photocured hydrogels with different PLMA mass fractions. (D) Cyclic compression profiles of photocured hydrogel specimens containing 10 and 15 wt% PLMA under repeated loading, supporting formulation optimization. (E) Predefined scaffold model and representative 3D-printed and projection-photocured HAMA, HP, and KGN@Lipo@HP hydrogel scaffolds, showing preserved macroscopic geometry and lattice architecture. Scale bar, 1 mm. (F) Overall printing-fidelity of the 3D-printed hydrogel scaffolds. (G) In situ photorheology tracking the storage modulus (G′) and loss modulus (G″) during 405-nm irradiation. (H) Swelling ratios of HAMA, HP, and KGN@Lipo@HP hydrogels. (I) Mechanical comparison of HAMA, HP, and KGN@Lipo@HP based on compression curves and modulus values. (J) FTIR spectra of HAMA, HP, and KGN@Lipo@HP. Data are presented as mean ± SD (n = 3) where applicable.

Having established the HP-associated cellular signature, the formulation, printability, and physicochemical properties underlying the scaffold platform was then examined. Fig. 2A illustrates the fabrication route, in which HAMA was hybridized with PLMA to form HP, followed by incorporation of KGN-loaded liposomes to generate KGN@Lipo@HP. Because PLMA served as the reinforcing and antibacterial component of the hybrid network, its mass fraction was first optimized. SEM analysis showed that increasing PLMA from 0 to 10 wt% progressively generated a more ordered lamellar porous architecture, whereas further increasing PLMA to 15 wt% increased pore-size heterogeneity (Fig. 2B). Quantitative analysis showed that PLMA incorporation enhanced the compressive modulus, while the 10 wt% formulation exhibited the lowest pore-size coefficient of variation among the tested groups (Fig. 2C). Complete compressive stress–strain curves and pore-size distribution data supporting this formulation selection are provided in Fig. S4. The 10 wt% PLMA composition was retained because it provided reinforcement while preserving the most uniform pore architecture, a feature relevant to stress distribution and compression stability.

Cyclic compression analysis was further performed to evaluate the repeated-loading behavior of PLMA-containing hydrogel specimens (Fig. 2D). The representative testing setup and programmed cyclic strain input are provided in Fig. S5. KGN@Lipo was characterized as the bioactive delivery module before incorporation into the optimized HP scaffold. As shown in Fig. S6, KGN@Lipo exhibited a vesicular morphology, a stable hydrodynamic size with a mildly negative zeta potential during storage, and a relatively narrow nanoscale size distribution. The loading performance of KGN@Lipo was further evaluated across three independently prepared batches. The EE remained above 98.9%, with an average value of approximately 99.02%, and the drug-loading capacity was approximately 12.2%, indicating efficient and reproducible KGN incorporation into the liposomal carrier. KGN release followed a gradual cumulative profile, supporting the use of liposomes as a local carrier for prolonged KGN presentation within the HP scaffold.

Using the optimized 10 wt% PLMA formulation, HAMA, HP, and KGN@Lipo@HP scaffolds were fabricated by projection-based photocuring 3D printing using the same workflow. The predefined scaffold model and representative printed constructs showed that all three formulations preserved their macroscopic geometry and lattice architecture after photocuring (Fig. 2E). Printing-fidelity analysis further confirmed high structural fidelity of the printed scaffolds (Fig. 2F). Additional representative photographs of the printed KGN@Lipo@HP scaffold before and after placement in the cartilage defect are provided in Fig. S7, and detailed printing parameters are summarized in Table S1. These results verify the feasibility of the HP-based formulations as projection-printable and photocurable bioinks.

In situ photorheology under 405 nm irradiation showed rapid gelation, as G′ quickly exceeded G″ and reached an elastic-dominant plateau (Fig. 2G). Supplementary rheological analysis further confirmed shear-thinning behavior and time-dependent viscosity stability of HAMA, HP, and KGN@Lipo@HP (Fig. S8B and C). Swelling analysis showed that PLMA incorporation reduced the swelling ratio relative to HAMA, whereas KGN@Lipo loading maintained a swelling level comparable to HP (Fig. 2H), indicating a more constrained yet adequately hydrated network. In vitro degradation analysis showed gradual mass loss over time in all groups, with KGN@Lipo@HP showing a degradation profile comparable to HP (Fig. S8A). This result indicates that liposomal loading did not destabilize the optimized HP network during degradation. Compression testing showed that HP exhibited higher compressive resistance and modulus than HAMA, while KGN@Lipo@HP remained mechanically comparable to HP (Fig. 2I), indicating that liposomal KGN loading preserved the optimized mechanical baseline. This improvement should nevertheless be interpreted against the depth dependence, anisotropy, and test-mode sensitivity of native articular cartilage [[17], [18], [19]]. Therefore, the present HP-based bioink can be positioned as a mechanically supportive and microenvironment-regulating scaffold for cartilage regeneration rather than an immediate load-bearing substitute for native cartilage.

FTIR spectra were consistent with the characteristic functional groups of the HP hybrid network (Fig. 2J). The broad absorption band at approximately 3300–3360 cm^−1^, observed in all formulations, was mainly assigned to O–H stretching from HAMA, with overlapping N–H contributions from the amide and amino groups of HAMA and PLMA. The feature near 1640 cm^−1^ was assigned to amide I (C=O stretching), whereas the ∼1540 cm^−1^ band corresponded to amide II (N-H bending/C-N stretching). These amide-related bands are present in both HAMA and PLMA, with an additional PLMA contribution in HP and KGN@Lipo@HP. Absorption across 1040-1150 cm^−1^ was mainly attributable to C-O-C/C-O stretching in the HAMA polysaccharide backbone. The hybrid spectrum also agreed with the PLMA reference in Fig. S9, consistent with PLMA incorporation. The similar principal band positions of HP and KGN@Lipo@HP further indicated that KGN@Lipo incorporation did not markedly alter the characteristic chemical features of the HP network.

### 3.3 Protective multi-omics of KGN@Lipo@HP relative to the control group

**Fig. 3:**
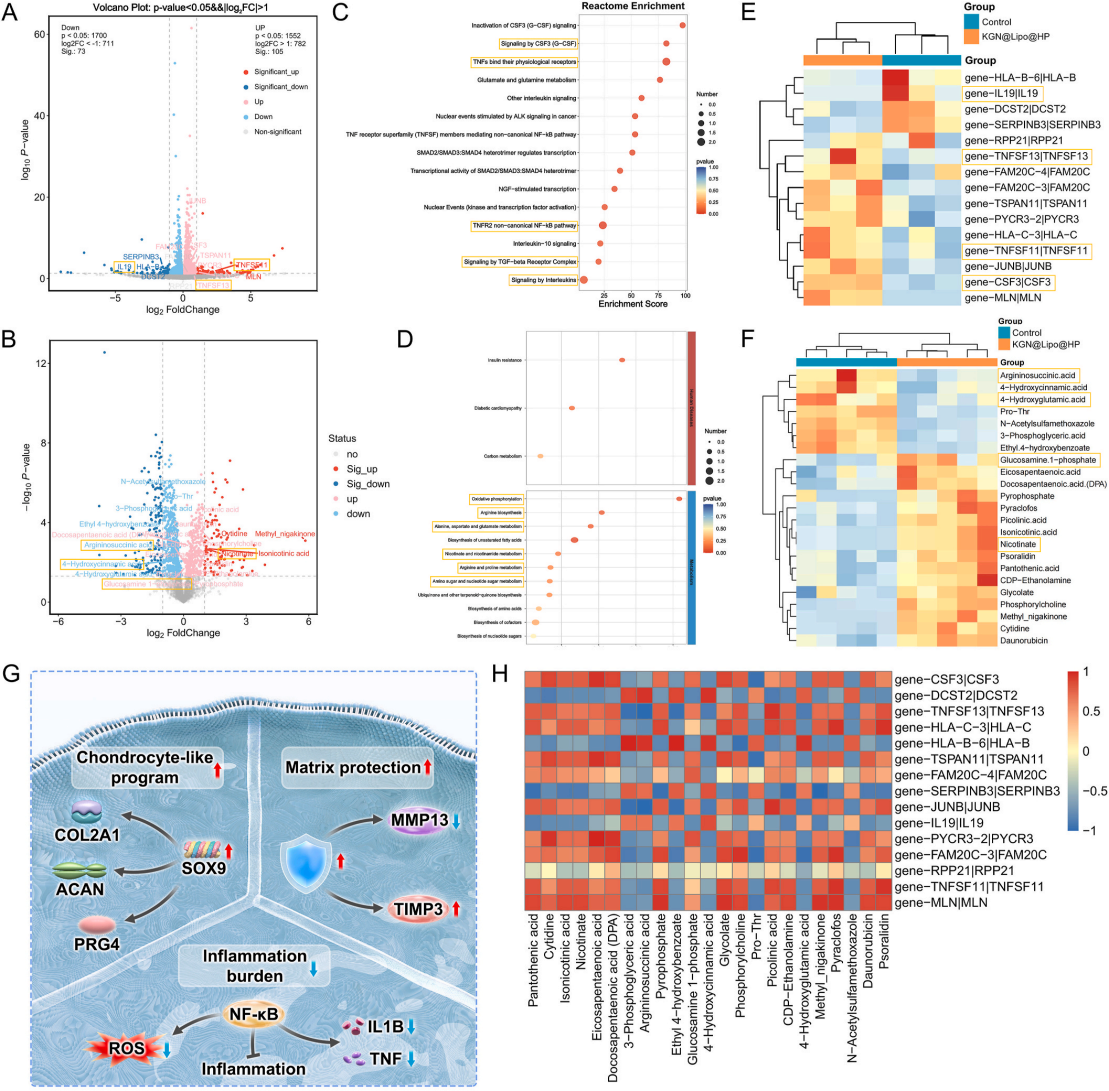
Protective multi-omics analysis of KGN@Lipo@HP relative to the Control group. (A) Transcriptomic differences between Control and KGN@Lipo@HP visualized in a volcano plot. (B) Metabolite differences between Control and KGN@Lipo@HP visualized in a volcano plot. (C) Reactome enrichment analysis of differentially expressed genes, highlighting cytokine-, interleukin-, TNF receptor-, TGF-β receptor-, and NF-κB-related regulatory pathways. (D) KEGG pathways enriched among differential metabolites, highlighting changes in oxidative phosphorylation, amino-acid metabolism, nicotinate/nicotinamide metabolism, and amino sugar and nucleotide sugar metabolism. (E,F) Hierarchical clustering heatmaps of representative differentially expressed genes (E) and metabolites (F). (G) Omics-supported working model linking KGN@Lipo@HP-associated signatures with chondrocyte-like anabolic remodeling, matrix protection, reduced ROS-associated stress, NF-κB-linked inflammatory signaling, and MMP13-associated catabolic remodeling. Arrows indicate proposed relationships within the omics-supported working model and do not denote experimentally established causal connections. (H) Integrated gene–metabolite correlation heatmap showing coordinated associations between differential genes and metabolites. Selected genes, metabolites, and pathway terms discussed in the text are highlighted in the corresponding panels for visual reference.

To characterize the systems-level cellular state associated with the final construct, a second integrated multi-omics comparison was performed between the Control and KGN@Lipo@HP groups. The transcriptomic volcano plot showed marked gene-expression divergence between the Control and KGN@Lipo@HP groups (Fig. 3A), and the metabolomic volcano plot likewise demonstrated substantial metabolite remodeling in response to the final construct (Fig. 3B).

Reactome enrichment linked the transcriptomic changes to cytokine- and inflammation-related pathways, including CSF3-associated signaling, TNF receptor-related pathways, interleukin signaling, TGF-β receptor complex regulation, and non-canonical NF-κB-related events (Fig. 3C). KEGG pathway analysis of the differential metabolites highlighted three amino acid–related pathways—arginine biosynthesis; alanine, aspartate and glutamate metabolism; and arginine and proline metabolism—together with oxidative phosphorylation, nicotinate and nicotinamide metabolism, and amino sugar and nucleotide sugar metabolism (Fig. 3D). Collectively, this pattern is consistent with metabolic adaptation accompanying inflammatory and signaling remodeling.

Hierarchical clustering of representative genes and metabolites further separated the Control and KGN@Lipo@HP groups (Fig. 3E and F). This pattern was reinforced by the supplementary metabolomic analyses, in which PCA, expanded differential-metabolite profiling, KEGG enrichment, and clustering all supported robust metabolite-level remodeling (Fig. S10). Complementary transcriptomic PCA, KEGG, and GO analyses similarly supported broad transcriptional reprogramming in response to the KGN@Lipo@HP microenvironment (Fig. S11).

Fig. 3G summarizes an omics-supported working model in which KGN@Lipo@HP is associated with a chondrocyte-like anabolic and matrix-protective signature, including SOX9-, COL2A1-, ACAN-, PRG4-, and TIMP3-related remodeling, together with reduced ROS-associated stress, NF-κB-linked inflammatory burden, and MMP13-associated catabolic signaling. The integrated transcriptome–metabolome analysis was consistent with this view, showing coordinated gene–metabolite associations (Fig. 3H), and the expanded interaction network and correlation heatmap in Fig. S12 further supported extensive systems-level connectivity. Together, the two omics comparisons define complementary material-associated cellular states: an HP-associated mechanotransduction signature and a matrix-protective state associated with the final KGN@Lipo@HP construct. Because these Control-referenced comparisons do not isolate the incremental contribution of KGN@Lipo, its added functional effect was subsequently examined through direct HP-versus-KGN@Lipo@HP comparisons in the assays described below. Accordingly, the omics data provide a systems-level framework for interpreting the observed phenotypes, while the functional assays provide independent phenotypic support for the material-associated outcomes. Causality at the level of individual molecular mediators will require targeted perturbation studies.

### 3.4 Cytoprotection and antibacterial activity

**Fig. 4:**
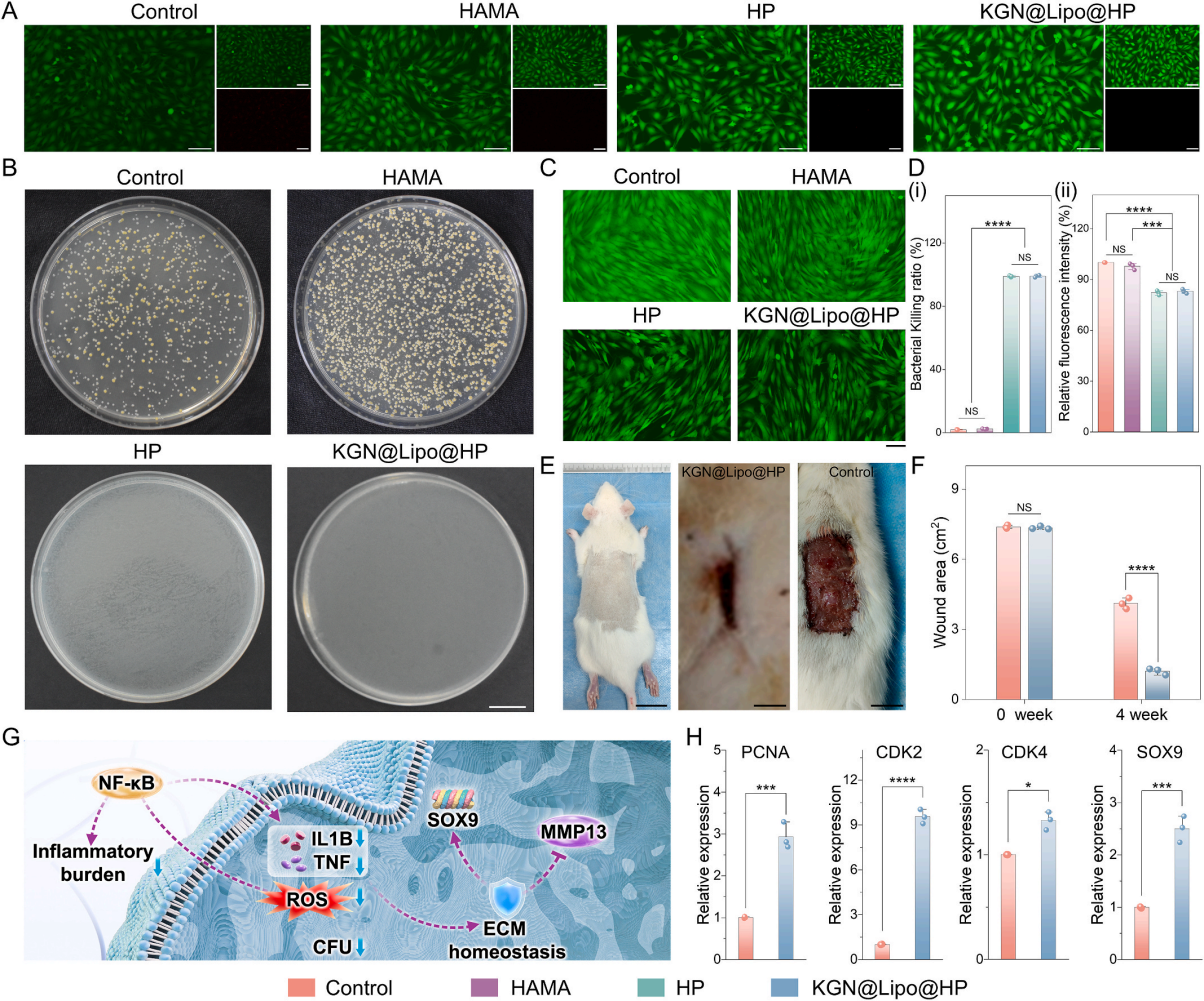
Cytocompatibility, oxidative-stress attenuation, antibacterial activity, and auxiliary rat cutaneous wound assessment of the hydrogel formulations. (A) Live/dead fluorescence micrographs for cells in the Control, HAMA, HP, and KGN@Lipo@HP conditions. Scale bar, 20 μm. (B) Representative *Staphylococcus aureus* colony-formation assays for the Control, HAMA, HP, and KGN@Lipo@HP groups. Scale bar, 1 cm. (C) DCFH-DA fluorescence imaging of intracellular ROS in the indicated groups. Scale bar, 20 μm. (D) Quantification of (i) the bacterial killing ratio and (ii) normalized DCFH-DA fluorescence. The two readouts were treated as separate datasets; each was analyzed by one-way ANOVA followed by Tukey's post hoc test. Values are mean ± SD (n = 3). (E) Representative images acquired at the endpoint of the auxiliary rat cutaneous-wound feasibility assay. Scale bars are 4 cm in the left panel and 1 cm in the middle and right panels. (F) Wound-area measurements at weeks 0 and 4 for the Control and KGN@Lipo@HP groups. Separate unpaired two-tailed Student's t-tests were applied at each time point; values are mean ± SD for three independent rats per group. (G) Integrated schematic summarizing the directly measured reductions in CFU and ROS-associated fluorescence, the omics-associated decreases in IL1B and TNF, and the qRT-PCR-detected increase in SOX9. Dashed arrows indicate literature-supported directional relationships, whereas dotted non-directional lines indicate crosstalk without assigning causal direction. The relationships involving ECM homeostasis, SOX9, and MMP13 represent an interpretive framework rather than pathway-specific causal conclusions. (H) qRT-PCR measurement of PCNA, CDK2, CDK4, and SOX9 mRNA in scaffold-free hBMSCs and cells co-cultured with KGN@Lipo@HP. Control conditions were assay-specific: no scaffold was used for the cell-based assays in panels A, C, D(ii), and H; scaffold-free PBS was used for the antibacterial assays in panels B and D(i); and no material treatment was applied in the rat wound assay in panels E and F. The groups were compared using an unpaired two-tailed Student's t-test, and values are mean ± SD (n = 3). NS, not significant; *p < 0.05; ***p < 0.001; ****p < 0.0001.

The protective state suggested by the omics analyses was then evaluated functionally. Live/dead staining showed abundant viable cells in all groups, indicating that neither PLMA incorporation nor KGN@Lipo loading introduced overt cytotoxicity into the hydrogel system (Fig. 4A). Antibacterial testing using PBS extracts prepared from the corresponding printed scaffolds showed dense colony formation in the Control and HAMA groups, whereas bacterial growth was markedly reduced in the HP and KGN@Lipo@HP groups (Fig. 4B). DCFH-DA imaging showed lower intracellular ROS-associated fluorescence in the HP and KGN@Lipo@HP groups than in the Control and HAMA groups (Fig. 4C). CFU-based killing-ratio analysis showed high antibacterial activity in the HP and KGN@Lipo@HP groups, whereas the Control and HAMA groups showed minimal bacterial killing (Fig. 4D(i)). The antibacterial effect is plausibly attributable to cationic PLMA: protonated amino groups in the polylysine-derived structure can bind electrostatically to negatively charged bacterial-membrane components, thereby limiting colonization [32]. Quantified DCFH-DA signals were lower in both HP-containing conditions than in Control and HAMA. No significant difference was detected between HP and KGN@Lipo@HP (Fig. 4D(ii)). An infected rat cutaneous wound assay was used as an auxiliary in vivo assessment of anti-infective feasibility. A representative week-0 image is provided in Fig. S13. Wound size did not differ at baseline. At week 4, the residual wound area was smaller in the KGN@Lipo@HP group than in the Control group (Fig. 4E and F).

Fig. 4G integrates the experimentally measured reductions in bacterial burden and ROS-associated fluorescence, the omics-associated decreases in IL1B and TNF, and the qRT-PCR-detected increase in SOX9 shown in Fig. 4H. The dashed and dotted connections place these findings within a literature-supported framework linking NF-κB-related inflammatory signaling and ROS-associated stress with cartilage ECM homeostasis, cartilage-anabolic responses, and MMP13-associated catabolic remodeling [5,21]. These relationships are presented as an interpretive framework rather than as pathway-specific causal conclusions of the present study. qRT-PCR analysis further showed increased mRNA expression of PCNA, CDK2, CDK4, and SOX9 in KGN@Lipo@HP relative to the Control group (Fig. 4H), indicating upregulation of proliferation- and chondrogenesis-related markers. The primer sequences used for qRT-PCR analysis of PCNA, CDK2, CDK4, SOX9, and the reference gene GAPDH are listed in Table S2. Collectively, these assays support the cytocompatibility and antibacterial activity of the formulations, together with reduced intracellular ROS-associated fluorescence and increased expression of proliferation- and chondrogenesis-related genes under the tested conditions.

### 3.5 In vitro chondrogenesis

**Fig. 5:**
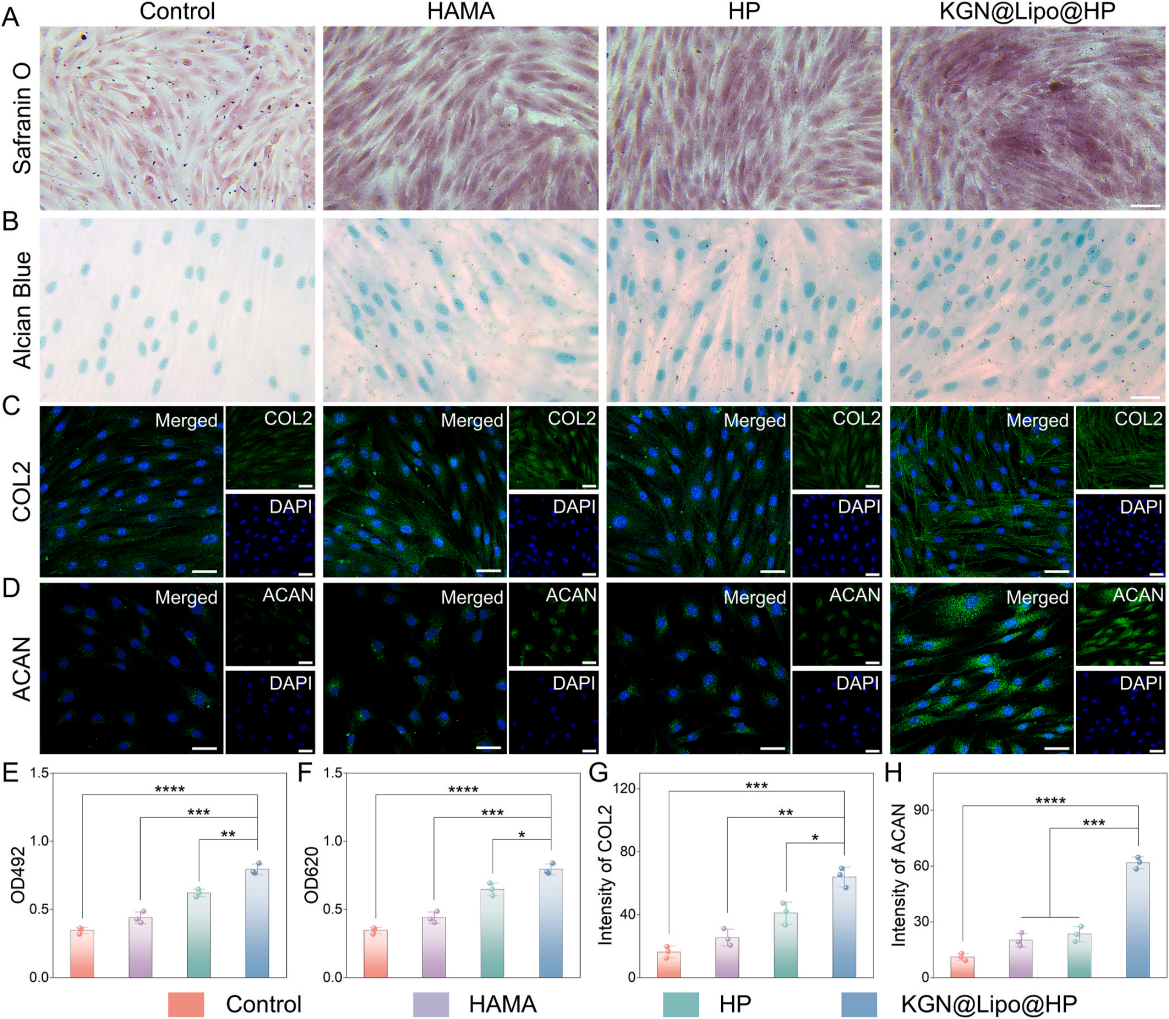
In vitro chondrogenesis and cartilage-matrix deposition induced by the hydrogel formulations. (A,B) Safranin O (A) and Alcian Blue (B) staining of the Control, HAMA, HP, and KGN@Lipo@HP groups. Scale bar, 20 μm. (C,D) COL2 (C) and ACAN (D) immunofluorescence, respectively, across the four groups; each panel includes a merged image and the corresponding single-channel views. Scale bar, 20 μm. (E,F) Semiquantitative optical-density values for Safranin O (E) and Alcian Blue (F), respectively. (G,H) Fluorescence-intensity measurements for COL2 (G) and ACAN (H), respectively. For all panels, Control denotes hBMSCs maintained in chondrogenic induction medium without a scaffold. Data are presented as mean ± SD (n = 3). Statistical significance was determined by one-way ANOVA followed by Tukey's post hoc test. *p < 0.05; **p < 0.01; ***p < 0.001; ****p < 0.0001. (For interpretation of the references to colour in this figure legend, the reader is referred to the Web version of this article.)

The effects of the material formulations on in vitro cartilage-matrix formation were next evaluated. Safranin O staining showed progressively stronger cartilage-associated matrix deposition from Control to HAMA and HP, with the strongest and most homogeneous staining in KGN@Lipo@HP (Fig. 5A). Alcian Blue staining followed the same trend, with the KGN@Lipo@HP group showing the most intense sulfated glycosaminoglycan-rich matrix deposition among all groups (Fig. 5B).

COL2 and ACAN immunofluorescence showed a corresponding protein-level pattern. COL2 staining was weakest in the Control group, increased in HAMA and HP, and reached its highest level in KGN@Lipo@HP (Fig. 5C), consistent with progressively greater cartilage-associated matrix deposition. ACAN showed the same pattern, with the strongest fluorescence signal in KGN@Lipo@HP (Fig. 5D), consistent with more robust deposition of cartilage-associated proteoglycan matrix.

The quantitative analyses matched the histological and immunofluorescent observations. Optical-density measurements of Safranin O and Alcian Blue confirmed that KGN@Lipo@HP yielded the highest matrix-staining values among all groups (Fig. 5E and F). Fluorescence-intensity quantification of COL2 and ACAN likewise showed that liposomal KGN loading on the optimized HP scaffold significantly enhanced cartilage-marker expression relative to the other formulations (Fig. 5G and H). Overall, mean Safranin O and Alcian Blue signals and COL2 and ACAN fluorescence were greater with HP than with HAMA. KGN@Lipo@HP yielded the highest values and reached significance in the comparisons shown in the figure. The HP-associated direction is therefore considered consistent with, rather than direct mechanistic proof of, part of the cartilage-anabolic working model. All quantitative analyses in Fig. 5 were based on three independent experiments (n = 3).

### 3.6 In vivo articular cartilage repair

**Fig. 6:**
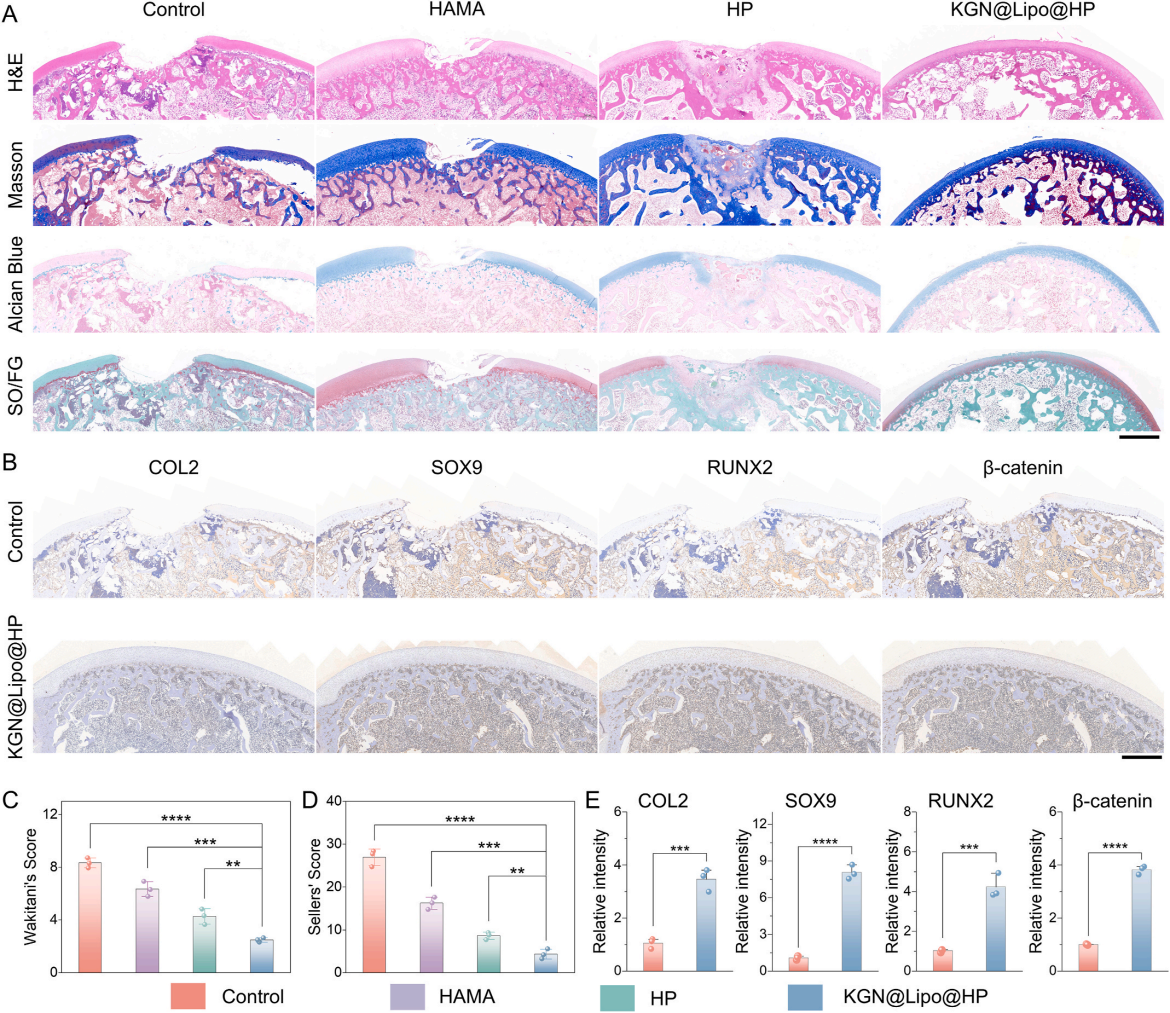
Rabbit articular cartilage repair induced by the hydrogel formulations. (A) Representative H&E, Masson, Alcian Blue, and SO/FG sections from Control defects (untreated and scaffold-free) and the HAMA, HP, and KGN@Lipo@HP groups. Scale bar, 1 mm. (B) Representative COL2, SOX9, RUNX2, and β-catenin immunohistochemistry for the Control and KGN@Lipo@HP groups. Scale bar, 1 mm. (C,D) Wakitani's score (C) and Sellers' score (D) for the four groups. Data are presented as mean ± SD (n = 3 independent rabbits per group). Statistical significance was determined by one-way ANOVA followed by Tukey's post hoc test. **p < 0.01; ***p < 0.001; ****p < 0.0001. (E) Relative immunohistochemical intensities of COL2, SOX9, RUNX2, and β-catenin in Control and KGN@Lipo@HP. For all panels, Control denotes untreated defects without scaffold implantation. Data are presented as mean ± SD (n = 3 independent rabbits per group). Statistical significance was determined by an unpaired two-tailed Student's t-test. *p < 0.05; ***p < 0.001; ****p < 0.0001. (For interpretation of the references to colour in this figure legend, the reader is referred to the Web version of this article.)

Finally, the in vivo reparative efficacy of KGN@Lipo@HP was assessed using a rabbit articular cartilage defect model. Control defects were left untreated without scaffold placement. Histology showed treatment-related variation in repair quality (Fig. 6A), and the KGN@Lipo@HP sections exhibited greater defect filling and better tissue continuity on H&E and Masson staining, while Alcian Blue and SO/FG staining indicated greater cartilage-like matrix deposition than in the Control, HAMA, and HP groups. High-magnification histological images are provided in Fig. S14 to further illustrate the repaired tissue morphology and matrix staining patterns. The KGN@Lipo@HP group had the lowest Wakitani and Sellers scores among the four groups, consistent with improved histological repair (Fig. 6C and D). Image-based morphometric analysis further showed that the cartilage-like matrix fill ratio was significantly higher in KGN@Lipo@HP than in the Control, HAMA, and HP groups, consistent with the histological scoring results (Fig. S14B).

Representative immunohistochemical staining illustrated treatment-associated differences in COL2, SOX9, RUNX2, and β-catenin between the Control and KGN@Lipo@HP groups (Fig. 6B). Semiquantitative analysis showed higher COL2 and SOX9 staining intensities in KGN@Lipo@HP than in Control, while RUNX2 and β-catenin staining intensities also differed significantly between the two groups (Fig. 6E). These findings indicate treatment-associated differences in cartilage-related and remodeling-associated marker profiles. Together, the concordant histological, scoring, and immunohistochemical findings support improved cartilage repair by KGN@Lipo@HP in the present rabbit model.

Two priorities for translation are to define the long-term relationship between scaffold degradation/remodeling and repair durability and to validate efficacy and safety in larger load-bearing animal models under clinically relevant joint-loading conditions. Subsequent scale-up will also require reproducible HP synthesis, batch-controlled KGN@Lipo loading, and sterilization-compatible processing.

## 4 Conclusion

A multi-omics-guided HP bioink incorporating KGN-loaded liposomes was developed for articular cartilage regeneration. Systems-level analyses first identified an HP-associated mechanotransduction and cartilage-anabolic signature in hBMSCs. Subsequent material characterization confirmed the printability, physicochemical stability, and mechanical reinforcement of the optimized HP platform and showed that KGN@Lipo incorporation preserved its structural and mechanical performance. The final KGN@Lipo@HP construct was associated with a matrix-protective multi-omics signature relative to the Control condition, while direct comparison with HP showed that incorporation of the KGN@Lipo module preserved scaffold mechanics and further enhanced cartilage-matrix formation and in vivo repair. Collectively, the material and biological data support KGN@Lipo@HP as a multifunctional bioink integrating mechanical support, intrinsic antibacteriality, and local KGN bioactivity. This work therefore provides a framework for designing 3D-printed bioinks that integrate manufacturability with microenvironmental regulation. Future studies using independently tunable matrices and pathway-specific perturbation may further define the stiffness-specific signaling mechanisms underlying cartilage regeneration.

## CRediT authorship contribution statement

**Tengfei Ma:** Data curation, Writing – original draft. **Peixuan Wu:** Investigation, Methodology. **Yongning Sheng:** Data curation. **Lian Duan:** Data curation. **Jie Pei:** Data curation. **Yanxin Liu:** Conceptualization, Data curation, Formal analysis, Investigation, Methodology, Resources, Supervision, Validation, Writing – review & editing. **Kun Fu:** Conceptualization, Data curation, Funding acquisition, Supervision. **Zeyu Liu:** Conceptualization, Data curation, Investigation, Methodology, Supervision, Validation, Visualization, Writing – original draft, Writing – review & editing.

## Declaration of competing interest

The authors declare no conflict of interest.

## Data Availability

Data will be made available on request.
