# Supplementary material for: Multi-omics-guided HAMA/PLMA bioink integrating mechanical support, antibacterial protection, and liposomal kartogenin for cartilage regeneration
Source: Mater Today Bio. 2026 Aug 10;40:103546. doi: 10.1016/j.mtbio.2026.103546 (PMC13486536; doi:10.1016/j.mtbio.2026.103546)
Supplement: Supplementary file 1 [file mmc1.docx]

**Supplementary Information**

**Multi-Omics-Guided HAMA/PLMA Bioink Integrating Mechanical Support, Antibacterial Protection, and Liposomal Kartogenin for Cartilage Regeneration**

*Tengfei Ma,^a,#^ Peixuan Wu,^b,#^ Yongning Sheng,^a^ Lian Duan,^c^ Jie Pei,^d^ Yanxin Liu,^e,^* Kun Fu,^a,f,^* Zeyu Liu ^g,^**

^a^ Department of Joint Surgery, The First Affiliated Hospital of Hainan Medical University, Haikou 570102, China

^b^ Key Laboratory of Advanced Materials of Tropical Island Resources of Ministry of Education, School of Chemical Engineering and Technology, Hainan University, Haikou 570228, China

^c^ Department of Comprehensive Surgery, Hainan Public Health Clinical Center, Haikou 570203, China

^d^ Department of Surgery, Haikou People's Hospital, Haikou 570208, China

^e^ Department of Bioengineering, University of California, Los Angeles, Los Angeles, California 90095, United States

^f^ Department of Joint Surgery, Hainan Sino-German Orthopedic Hospital, Haikou 570200, China

^g^ Center for Musculoskeletal Surgery, Charité–Universitätsmedizin Berlin, Berlin 13353, Germany

*^#^* These authors contributed equally to this work.

* Corresponding authors

Email: [Liuyanxin2121@163.com](mailto:Liuyanxin2121@163.com) (Y. Liu), [fukun2015@126.com](mailto:fukun2015@126.com) (K. Fu), [zeyu.liu@charite.de](mailto:zeyu.liu@charite.de) (Z. Liu)
**Abbreviations:** HAMA, hyaluronic acid methacrylate; PLMA, polylysine methacrylamide; HP, HAMA/PLMA hydrogel scaffold; KGN, kartogenin; KGN@Lipo, KGN-loaded liposomes; KGN@Lipo@HP, KGN@Lipo-loaded HP scaffold; hBMSC, human bone marrow mesenchymal stem cell; ECM, extracellular matrix; ROS, reactive oxygen species; DCFH-DA, 2′,7′-dichlorodihydrofluorescein diacetate; CFU, colony-forming units; EE, encapsulation efficiency; DL, drug-loading capacity; SEM, scanning electron microscopy; FTIR, Fourier-transform infrared spectroscopy; qRT-PCR, quantitative real-time polymerase chain reaction; H&E, hematoxylin and eosin; SO/FG, Safranin O/Fast Green; SD, standard deviation; PCA, principal component analysis; KEGG, Kyoto Encyclopedia of Genes and Genomes; GO, Gene Ontology; NS, not significant.

Supplementary Methods

1. Transcriptomic data processing and analysis

Raw sequencing reads were filtered using fastp (v0.23.4). Adapter-contaminated reads were removed; bases were trimmed using a four-base sliding window when the mean Phred quality score fell below Q20; reads containing more than five undetermined bases were excluded; and reads shorter than 15 nt were discarded. Clean paired-end reads were aligned to the human reference genome GRCh38.p14 (GCF_000001405.40) with Ensembl release 110 annotation using HISAT2 (v2.2.1). StringTie2 (v2.1.4) was used to reconstruct transcripts and quantify gene expression. FPKM and TPM values were generated for expression reporting, whereas raw gene counts were used for differential expression analysis. Count data were normalized using the DESeq2 size-factor method, and differential expression analysis was performed using DESeq2 (v1.38.3). Differentially expressed genes were defined by |log_2_FoldChange| > 1 and adjusted p value (padj) < 0.05. Principal component analysis and hierarchical clustering were performed using the normalized expression matrix. Gene Ontology, KEGG, and Reactome enrichment analyses were conducted using clusterProfiler (v4.6.2), with padj < 0.05 considered significant. The Control-versus-HP and Control-versus-KGN@Lipo@HP comparisons were processed using the same workflow.

2. Metabolomic data processing and analysis

Untargeted metabolomic profiling was performed using a Vanquish UHPLC system coupled to an Orbitrap Exploris 120 mass spectrometer. Raw data were converted to mzXML format using ProteoWizard (v3.0.24054) and processed using an in-house R workflow based on XCMS (v4.1.12) for peak detection, retention-time alignment, peak integration, and feature extraction. Metabolites were annotated using BiotreeDB (v3.0). Features were retained when the relative standard deviation in QC samples was < 0.30 and they were detected in at least 50% of samples in at least one experimental group. Pairwise correlations among QC samples were required to be > 0.90. Missing values were imputed using the half-minimum method, and signal intensities were normalized by total ion current. No additional transformation or scaling procedure was documented in the available analysis records. Principal component analysis was used to evaluate sample clustering. OPLS-DA was performed using SIMCA (v16.0.2), and VIP values were derived from the corresponding OPLS-DA model for each group comparison. Differential metabolites were defined by VIP > 1, Student's t-test p < 0.05, and fold change >= 2 or <= 0.5. KEGG pathway-enrichment analysis was performed for the differential metabolites. The same preprocessing and differential-analysis workflow was applied to the Control-versus-HP and Control-versus-KGN@Lipo@HP comparisons.

3. Integrated transcriptome–metabolome analysis

Differentially expressed genes and differential metabolites were integrated based on shared pathway annotations and gene-metabolite correlations. Spearman correlation coefficients were calculated for gene-metabolite pairs. Correlation-network edges were retained using |r| > 0.8 and p < 0.05, without FDR correction. Correlation heatmaps were generated using the R package pheatmap (v1.0.12), and correlation networks were generated in R (v4.3.3); Cytoscape was not used. Integrated analyses were performed separately for the Control-versus-HP and Control-versus-KGN@Lipo@HP comparisons.

Table S1. Printing parameters used for 3D scaffold fabrication.

| **Parameter** | **Value** |
| --- | --- |
| Printer | EFL-BP8601pro, EFL, Suzhou, China |
| Printing mode | Projection-based photocuring 3D printing |
| Bioink formulations | HAMA, HP, KGN@Lipo@HP |
| HAMA concentration | 10 wt% |
| LAP concentration | 0.25 wt% |
| PLMA content in optimized HP | 10 wt% |
| Final KGN concentration in KGN@Lipo@HP | 100 μM |
| Designed pore interspace | 500 μm |
| Designed scaffold geometry | Cylindrical porous scaffold |
| Designed scaffold diameter | 5 mm |
| Designed scaffold thickness | 3 mm |
| Photocuring wavelength | 405 nm |
| Light intensity | 30 mW cm⁻² |
| Layer thickness | 100 μm |
| Exposure time | 30 s |
| Post-curing | 5 s |
| Pre-use treatment | PBS rinse and culture medium equilibration overnight |
| Printing-fidelity imaging | Stereomicroscopy / optical microscopy |
| Fidelity metrics | Pore interspace, pore-size coefficient of variation, scaffold diameter, scaffold thickness, and dimensional error |

Table S2. Primer sequences used for qRT-PCR analysis.

| **Primer name** | **Orientation** | **Sequence (5′–3′)** |
| --- | --- | --- |
| PCNA | Forward | CAAGTAATGTCGATAAAGAGGAGG |
| PCNA | Reverse | GTGTCACCGTTGAAGAGAGTGG |
| CDK2 | Forward | ATGGATGCCTCTGCTCTCACTG |
| CDK2 | Reverse | CCCGATGAGAATGGCAGAAAGC |
| CDK4 | Forward | CCATCAGCACAGTTCGTGAGGT |
| CDK4 | Reverse | TCAGTTCGGGATGTGGCACAGA |
| SOX9 | Forward | AGGAAGCTCGCGGACCAGTAC |
| SOX9 | Reverse | GGTGGTCCTTCTTGTGCTGCAC |
| GAPDH | Forward | GGAGCGAGATCCCTCCAAAAT |
| GAPDH | Reverse | GGCTGTTGTCATACTTCTCATGG |
| ITGB1 | Forward | TGTTCAGTGCAGAGCCTTCA |
| ITGB1 | Reverse | CCTCATACTTCGGATTGACCAC |
| PTK2 | Forward | GGCATCATTCAGAAGATAGTGG |
| PTK2 | Reverse | CCACAAACTCCTCATCTTCATT |
| ROCK1 | Forward | AACATGCTGCTGGATAAATCTGG |
| ROCK1 | Reverse | TGTATCACATCGTACCATGCCT |
| YAP1 | Forward | TAGCCCTGCGTAGCCAGTTA |
| YAP1 | Reverse | TCATGCTTAGTCCACTGTCTGT |
| WWTR1 | Forward | GGTGATGGAAGATGACCTGG |
| WWTR1 | Reverse | GCTGCTCATAGGGCTTGTTT |
| CCN2 | Forward | CAGCATGGACGTTCGTCTG |
| CCN2 | Reverse | AACCACGGTTTGGTCCTTGG |

Table S3. Exact p values for quantitative comparisons shown in the figures.

| **Figure** | **Panel** | **Comparison** | **Parameter** | **Exact p value** |
| --- | --- | --- | --- | --- |
| Figure 4 | D(i) | Control vs KGN@Lipo@HP | Quantitative analysis of bacterial killing ratio | p = 8.183◊10^-10^ |
| Figure 4 | D(i) | Control vs HP | Quantitative analysis of bacterial killing ratio | p = 2.12◊10^-9^ |
| Figure 4 | D(i) | HAMA vs KGN@Lipo@HP | Quantitative analysis of bacterial killing ratio | p = 2.411◊10^-9^ |
| Figure 4 | D(i) | HAMA vs HP | Quantitative analysis of bacterial killing ratio | p = 4.394◊10^-6^ |
| Figure 4 | D(ii) | Control vs KGN@Lipo@HP | Relative fluorescence intensity | p = 2.116◊10^-5^ |
| Figure 4 | D(ii) | Control vs HP | Relative fluorescence intensity | p = 2.242◊10^-5^ |
| Figure 4 | D(ii) | HAMA vs KGN@Lipo@HP | Relative fluorescence intensity | p = 3.138◊10^-4^ |
| Figure 4 | D(ii) | HAMA vs HP | Relative fluorescence intensity | p = 3.772◊10^-4^ |
| Figure 4 | F | Control vs KGN@Lipo@HP | Wound-area quantification | p = 6.249◊10^-5^ |
| Figure 4 | H | Control vs KGN@Lipo@HP | Relative expression of PCNA | p = 9.727◊10^-4^ |
| Figure 4 | H | Control vs KGN@Lipo@HP | Relative expression of CDK2 | p = 4.394◊10^-6^ |
| Figure 4 | H | Control vs KGN@Lipo@HP | Relative expression of CDK4 | p = 1.127◊10^-2^ |
| Figure 4 | H | Control vs KGN@Lipo@HP | Relative expression of SOX9 | p = 1.248◊10^-4^ |
| Figure 5 | E | Control vs KGN@Lipo@HP | OD492 | p = 9.673◊10^-4^ |
| Figure 5 | E | HAMA vs KGN@Lipo@HP | OD492 | p = 2.215◊10^-3^ |
| Figure 5 | E | HP vs KGN@Lipo@HP | OD492 | p = 1.089◊10^-2^ |
| Figure 5 | F | Control vs KGN@Lipo@HP | OD620 | p = 4.168◊10^-5^ |
| Figure 5 | F | HAMA vs KGN@Lipo@HP | OD620 | p = 5.298◊10^-4^ |
| Figure 5 | F | HP vs KGN@Lipo@HP | OD620 | p = 3.185◊10^-2^ |
| Figure 5 | G | Control vs KGN@Lipo@HP | Intensity of COL2 | p = 7.597◊10^-4^ |
| Figure 5 | G | HAMA vs KGN@Lipo@HP | Intensity of COL2 | p = 1.588◊10^-3^ |
| Figure 5 | G | HP vs KGN@Lipo@HP | Intensity of COL2 | p = 4.182◊10^-2^ |
| Figure 5 | H | Control vs KGN@Lipo@HP | Intensity of ACAN | p = 1.899◊10^-5^ |
| Figure 5 | H | HAMA vs KGN@Lipo@HP | Intensity of ACAN | p = 1.096◊10^-4^ |
| Figure 5 | H | HP vs KGN@Lipo@HP | Intensity of ACAN | p = 1.096◊10^-4^ |
| Figure 6 | C | Control vs KGN@Lipo@HP | Wakitani’s score | p = 1.682◊10^-5^ |
| Figure 6 | C | HAMA vs KGN@Lipo@HP | Wakitani’s score | p = 3.551◊10^-4^ |
| Figure 6 | C | HP vs KGN@Lipo@HP | Wakitani’s score | p = 7.142◊10^-3^ |
| Figure 6 | D | Control vs KGN@Lipo@HP | Sellers’ score | p = 6.802◊10^-5^ |
| Figure 6 | D | HAMA vs KGN@Lipo@HP | Sellers’ score | p = 3.671◊10^-4^ |
| Figure 6 | D | HP vs KGN@Lipo@HP | Sellers’ score | p = 6.762◊10^-3^ |
| Figure 6 | E | Control vs KGN@Lipo@HP | Relative intensity of COL2 | p = 8.536◊10^-4^ |
| Figure 6 | E | Control vs KGN@Lipo@HP | Relative intensity of SOX9 | p = 3.684◊10^-5^ |
| Figure 6 | E | Control vs KGN@Lipo@HP | Relative intensity of RUNX2 | p = 8.659◊10^-4^ |
| Figure 6 | E | Control vs KGN@Lipo@HP | Relative intensity of β-catenin | p = 6.886◊10^-6^ |

Table S4. Mechanotransduction-associated markers selected for qRT-PCR validation.

| **Gene** | **Common name** | **Mechanotransduction module** | **Biological relevance in this study** |
| --- | --- | --- | --- |
| ITGB1 | Integrin β1 | ECM sensing / integrin-mediated adhesion | Represents cell–matrix adhesion and ECM mechanical-cue sensing. |
| PTK2 | FAK | Focal adhesion signaling | Encodes focal adhesion kinase, a key mediator linking integrin engagement to downstream mechanotransduction. |
| ROCK1 | ROCK1 | Rho/ROCK-mediated cytoskeletal tension | Reflects cytoskeletal contractility and actomyosin-associated mechanosensing. |
| YAP1 | YAP | Hippo/YAP mechanotransduction | Represents a mechanosensitive transcriptional co-activator downstream of cytoskeletal tension. |
| WWTR1 | TAZ | Hippo/TAZ mechanotransduction | Encodes TAZ, the paralog of YAP, and reflects YAP/TAZ-associated mechanotransduction at the transcriptional level. |
| CCN2 | CTGF | YAP/TAZ downstream transcriptional output / ECM remodeling | A canonical YAP/TAZ-responsive downstream marker associated with ECM remodeling and mechanotransductive transcriptional output. |

Figure S1. Supplementary metabolomic analyses comparing the Control and HP groups. (A) Volcano plot of differential metabolites between the Control and HP groups. (B) Principal component analysis (PCA) showing clear metabolic separation between the two groups. (C) KEGG enrichment analysis of differential metabolites. (D) Hierarchical clustering heatmap of representative metabolites.

Figure S2. Supplementary integrated transcriptome–metabolome analyses of the Control and HP groups. (A) Gene–metabolite association network showing the integrated relationships between differential genes and metabolites. Squares represent metabolites and circles represent genes; red indicates upregulation and blue indicates downregulation. (B) Correlation heatmap showing coordinated associations between representative differential genes and metabolites.

Figure S3. qRT-PCR validation of mechanotransduction-associated markers. Relative mRNA expression levels of ITGB1, PTK2, ROCK1, YAP1, WWTR1, and CCN2 in hBMSCs co-cultured with the indicated scaffolds. Data are presented as mean ± SD (n = 3). Statistical significance was determined by one-way ANOVA followed by Tukey’s post hoc test. Statistical annotations are defined in the main-text Statistics section.

Figure S4. Supplementary characterization for HP hydrogels with 0, 5, 10, and 15 wt% PLMA. (A) Pore-size distribution. (B) Compressive stress–strain curves.

Figure S5. Representative cyclic compression test setup and loading protocol. (A) Representative photograph showing the hydrogel specimen positioned between the compression platens during testing. (B) Programmed cyclic strain input used for cyclic compression testing, consisting of repeated 0–20% strain cycles over 150 s.

Figure S6. Physicochemical characterization, loading performance, and release behavior of KGN@Lipo. (A) Representative image showing the vesicular morphology of KGN@Lipo. Scale bar, 50 nm. (B) Hydrodynamic size and zeta potential of KGN@Lipo during storage. (C) Size-distribution profile of KGN@Lipo. (D) Cumulative release profile of KGN from KGN@Lipo over time. (E) Encapsulation efficiency and drug-loading capacity of KGN@Lipo from three independently prepared batches. Each batch was measured in triplicate.

Figure S7. Representative photographs of projection-based 3D-printed and photocured KGN@Lipo@HP hydrogel scaffolds. (A) Top-view optical image showing the printed porous lattice architecture. Scale bar, 0.5 mm. (B) Macroscopic photograph of the printed scaffold. Scale bar, 2.5 mm. (C) Representative intraoperative image showing the printed scaffold immediately after placement in the rabbit cartilage defect. Scale bar, 7 mm.

Figure S8. Supplementary degradation and rheological characterization of HAMA, HP, and KGN@Lipo@HP. (A) In vitro degradation profiles of HAMA, HP, and KGN@Lipo@HP hydrogels, shown as weight remaining over time. (B) Viscosity as a function of shear rate, showing pronounced shear-thinning behavior of the three formulations. (C) Time-dependent viscosity profiles of HAMA, HP, and KGN@Lipo@HP.

Figure S9. FTIR spectrum of PLMA.

Figure S10. Supplementary metabolomic analyses comparing the Control and KGN@Lipo@HP groups. (A) Principal component analysis (PCA) showing clear metabolic separation between the two groups. (B) Volcano plot of differential metabolites between the Control and KGN@Lipo@HP groups. (C) KEGG enrichment analysis of differential metabolites. (D) Hierarchical clustering heatmap of representative metabolites.

Figure S11. Supplementary transcriptomic analyses comparing the Control and KGN@Lipo@HP groups. (A) Principal component analysis (PCA) showing clear transcriptomic separation between the two groups. (B) KEGG enrichment analysis of differentially expressed genes. (C) GO enrichment analysis of differentially expressed genes.

Figure S12. Supplementary integrated transcriptome–metabolome analyses of the Control and KGN@Lipo@HP groups. (A) Gene–metabolite association network showing the integrated relationships between differential genes and metabolites. Squares represent metabolites and circles represent genes; red indicates upregulation and blue indicates downregulation. (B) Correlation heatmap showing coordinated associations between representative differential genes and metabolites.

Figure S13. Representative image of the auxiliary rat cutaneous wound assay at week 0. Scale bar, 1 cm.

Figure S14. High-magnification histological images of repaired rabbit cartilage defects. (A) High-magnification Masson, Alcian Blue, and Safranin O/Fast Green staining images of defect sections from the Control, HAMA, HP, and KGN@Lipo@HP groups. Scale bar, 1 mm. (B) Cartilage-like matrix fill ratio quantified from SO/FG-stained sections. Data are presented as mean ± SD (n = 3 independent rabbits per group). Statistical significance was determined by an unpaired two-tailed Student’s t-test. ***p < 0.001; ****p < 0.0001.
